# High prevalence of bronchiectasis is linked to HTLV-1-associated inflammatory disease

**DOI:** 10.1186/s12879-015-1002-0

**Published:** 2015-07-06

**Authors:** Shohreh Honarbakhsh, Graham P Taylor

**Affiliations:** National Centre for Human Retrovirology, St Mary’s Hospital, Imperial College Healthcare NHS Trust, Praed St, W2 1NY London, UK; Section of Infectious Diseases, Department of Medicine, Imperial College London, London, UK

**Keywords:** Human T-lymphotropic virus type 1, Bronchiectasis, Inflammation

## Abstract

**Background:**

Human T-lymphotropic virus type 1 (HTLV-1), a retrovirus, is the causative agent of HTLV-1-associated myelopathy/tropical spastic paraparesis (HAM/TSP) and adult T-cell leukaemia/lymphoma (ATLL). The reported association with pulmonary disease such as bronchiectasis is less certain.

**Methods:**

A retrospective case review of a HTLV-1 seropositive cohort attending a national referral centre. The cohort was categorised into HTLV-1 symptomatic patients (SPs) (ATLL, HAM/TSP, Strongyloidiasis and HTLV associated inflammatory disease (HAID)) and HTLV-1 asymptomatic carriers (ACs). The cohort was reviewed for diagnosis of bronchiectasis.

**Result:**

34/246 ACs and 30/167 SPs had been investigated for respiratory symptoms by computer tomography (CT) with productive cough +/- recurrent chest infections the predominant indications. Bronchiectasis was diagnosed in one AC (1/246) and 13 SPs (2 HAID, 1 ATLL, 10 HAM/TSP) (13/167, RR 19.2 95 % CI 2.5-14.5, p = 0.004) with high resolution CT. In the multivariate analysis ethnicity (p = 0.02) and disease state (p < 0.001) were independent predictors for bronchiectasis. The relative risk of bronchiectasis in SPs was 19.2 (95 % CI 2.5-14.5, p = 0.004) and in HAM/TSP patients compared with all other categories 8.4 (95 % CI 2.7-26.1, p = 0.0002). Subjects not of African/Afro-Caribbean ethnicity had an increased prevalence of bronchiectasis (RR 3.45 95 % 1.2-9.7, p = 0.02).

**Conclusions:**

Bronchiectasis was common in the cohort (3.4 %). Risk factors were a prior diagnosis of HAM/TSP and ethnicity but not HTLV-1 viral load, age and gender. The spectrum of HTLV-associated disease should now include bronchiectasis and HTLV serology should be considered in patients with unexplained bronchiectasis.

## Author summary

The spectrum of disease associated with HTLV-1 has not been completely defined. The association between HTLV-1 and pulmonary conditions is less well documented and clinically recognised. Bronchiectasis has been reported amongst Australian aborigines infected with HTLV-1. We have observed a high rate of bronchiectasis in a UK HTLV-1 infected cohort. We have shown that patients with non-pulmonary HTLV-1-associated disease, particularly inflammatory disease, are more likely to have an additional diagnosis of bronchiectasis than asymptomatic carriers and postulate that the same dysregulated hyper-inflammatory response seen in HTLV-associated myelopathy/tropical spastic paraparesis may play a role in this setting. Our findings highlight the importance of screening HTLV-1 infected patients, particularly those with inflammatory disease, for pulmonary disease.

## Background

The human T-lymphotropic virus type-1 (HTLV-1), a retrovirus, has been estimated to infect at least 5-10 million people worldwide with the highest prevalence reported in Japan, Africa, The Caribbean and South America [1]. It is the causative agent of adult T-cell leukaemia/lymphoma (ATLL) [2], with a life-time risk of 3 – 7 % [3], of HTLV-1-associated myelopathy/tropical spastic paraparesis (HAM/TSP) [4, 5] affecting up to 4 % of carriers [6] and of HTLV-1-associated uveitis [7]. However the full spectrum and risk of developing a HTLV-1-associated disease remains uncertain with a much broader list of affected systems reported including: polymyositis [8], arthropathy [9], Sjögren’s syndrome (keratoconjunctivitis sicca) [10, 11], thyroiditis [12] and infective dermatitis [13]. It is understood that HAM/TSP is secondary to a dysfunctional inflammatory response [14] to high HTLV-1 pro-viral load (VL) [15, 16] and although less studied a similar relationship between other inflammatory conditions (uveitis [17], polymyositis [18]) and VL has been observed.

Although less recognised associations between HTLV-1 and pulmonary conditions have been reported: patients with HTLV-1 have increased lymphocytes in bronchoalveolar lavage fluid (BAL) which correlates with HTLV-1 VL in both the BAL and peripheral blood mononuclear cells (PBMC) [19]; patients with HAM/TSP [20] or HTLV-1-associated uveitis [21] have been shown to have significantly higher BAL lymphocyte counts than HTLV asymptomatic carriers (ACs); histology of surgical or transbronchial biopsies has revealed lymphocytic infiltration along bronchioles and bronchovascular bundles [22]; IL-2 receptor levels are markedly elevated in BAL from patients with HAM/TSP compared to ACs [23]; high frequency of activated T cells and increased MIP-1alpha and IP-10 concentrations have been found in BAL from patients with HTLV-1 and cryptogenic fibrosing alveolitis [24].

To date the most comprehensive report of pulmonary imaging in HTLV-1 infection is that of Okado *et al. *[22] who undertook a retrospective review of computerised tomography (CT) lung studies of 320 patients. Abnormalities, predominantly parenchymal, were reported in 98 patients (30.1 %) of which almost all had centrilobular nodules (95/320) and half had thickening of bronchovascular bundles (55/320), ground glass opacities (51/320) and/or bronchiectasis (50/320; 15.6 %). The indications for CT are not detailed but in almost 1/3 the scan was performed prior to the diagnosis of HTLV-1 infection. Even higher rates (61.3 %) but similar abnormalities among HTLV-1 infected ACs were reported from Okinawa [25]. Two additional studies of indigenous Australians have shown an association between HTLV-1 infection and bronchiectasis [26, 27]. A central Australian case-control study reported an association between HTLV-1 VL and bronchiectasis. Despite much higher VL in the cases than the matched controls this association is not maintained in the multivariate analysis [26]. Further to this in a retrospective study of indigenous Australians with bronchiectasis HTLV-1 infection was associated with more extensive disease and more bronchiectasis-related deaths (OR 5.78 95 % CI 1.17 - 26.75, p 0.028) [28]. A causal association between HTLV-1 and bronchiectasis has not been proven and it is important to note that outside of Japan reports relating to bronchiectasis concern Australian aborigines living in Central Australia who are known to have distinct socio-economic circumstances and health concerns that also differ from many HTLV-1 infected populations. Significantly in a prospective cohort study that included 152 HTLV-1 infected blood donors in the USA followed for a median of 4.4 years no increase in respiratory infections was found compared with the uninfected blood donors [29]. It is therefore uncertain whether confounders exist in either population which together with, or separately from, HTLV-1 predispose to bronchiectasis. Given the association between HTLV-1 VL and HTLV-1 associated diseases, whether inflammatory, infectious or malignant we postulated that if HTLV-1 is associated with an increased risk of bronchiectasis, this would be more common in patients with high HTLV-1 VL. We therefore conducted a retrospective study to describe the prevalence of, and factors associated with, a formal diagnosis of bronchiectasis.

Although HTLV-1 associated diseases are rare in the UK the National Centre for Human Retrovirology manages a relatively large HTLV cohort, the only one of its kind in Europe. The cohort was initially established in 1993 to gain an understanding of the breadth and pathogenesis of HTLV disease through longitudinal observation. Despite the aforementioned reports HTLV-1 infected patients are not generally considered to be at greater risk of bronchiectasis than the general population and therefore chest imaging in our cohort has only been conducted if there was a clinical suspicion of lung disease.

## Methods

A single national centre retrospective case review of all 464 HTLV-1 seropositive patients attending the National Centre for Human Retrovirology, St Mary’s Hospital, London, UK between 1993 and 2012. Subjects were characterised in regard to HTLV-1 infection as either symptomatic patients (SP) or as ACs. SPs had an underlying disease with documented association with HTLV-1: ATLL; strongyloidiasis; HAM/TSP; or other HTLV associated inflammatory disease (HAID) namely polymyositis, uveitis and arthritis. Twenty-three patients were excluded as they were co-infected with human immunodeficiency virus type 1. A further 28 patients were excluded as they had neurological features that could not be classified as either HAM/TSP or polymyositis and it was difficult to establish whether HTLV-1 infection was causal or co-incidental and thus whether they had underlying HTLV-1-associated disease.

The clinic database, case notes, laboratory and radiology reports were examined. In accordance with the guidance produced by the UK National Research Ethics Service, research ethics approval was not required for this study. Once the data were collated we used the clinical code, which is separate from the hospital number, to ensure confidentiality. Bronchiectasis was correlated with HTLV-1 symptoms and with concurrent HTLV-1 VL (this being the number of HTLV-1 DNA copies/100 PBMCs and reported as VL %). Data were collated on the number of patients that had CT i.e. CT chest or high resolution CT (HRCT), the clinical indication for the imaging and the radiological findings particularly the presence or absence of bronchiectasis. A multi-detector CT scanner was used. The CT criteria that were used for diagnosis of bronchiectasis were bronchial dilatation as evidence by bronchoarterial ratio greater than 1 to 1.5 (signet ring sign) and/or non-tapering airways (airways visible within 1 cm of the visceral pleura, tramlines and flaring). Indirect signs such as thickening of the bronchial wall, loss of lobe volume, mosaic perfusion pattern, a tree-in- bud pattern, and mucus plugs were also used.

Data was also collected on risk factors for bronchiectasis i.e. congenital conditions, tuberculosis, childhood infection and exposure to chemical irritant. We also collected data on the presence of other chronic conditions i.e. chronic liver disease, myocardial disease, renal and cardiac failure. Data were analysed by Student *t*-test, relative risk (RR), Fisher’s exact test and backwards multivariate logistic regression using commercial statistics software (SPSS 21, SPSS Inc, Chicago). A p-value of 0.05 or less was considered significant.

## Results

**Fig. 1:**
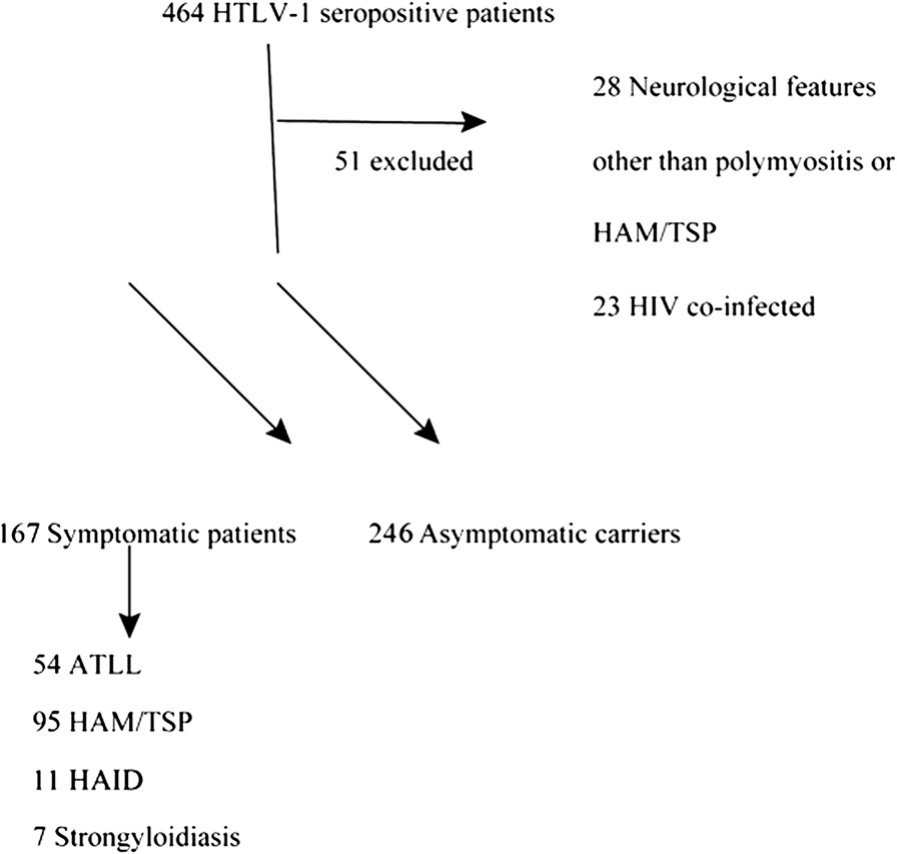
Flow diagram demonstrating the breakdown of the HTLV-1 cohort into symptomatic patients and asymptomatic carriers

Four hundred and thirteen patients infected with HTLV-1 were included comprising 246 ACs and 167 SPs. Of the 167 SPs: 54 had ATLL; 95 HAM/TSP; 11 HAID and 7 strongyloidiasis (Fig. 1). Fourteen (3.4 %), were found on pulmonary CT to have previously undiagnosed bronchiectasis.

Although the majority of all subjects were of African/Afro-Caribbean origin (300/413, 72.6 %), SPs were significantly more likely to be of African/Afro-Caribbean ethnicity (139/167, 83.2 %) than the ACs (161/246, 65.5 %, p < 0.0001) (Table 1) and this applied across all the symptomatic subgroups: HAM/TSP 74/95 (77.9 %); ATLL 48/54 (88.9 %); Strongyloidiasis 7/7 (100 %); HAID 10/11 (91 %). SPs were older than ACs (median age 54 vs. 44 years; p < 0.0001) but there was no difference in the time since HTLV-1 was diagnosed (i.e. duration of follow-up from first diagnosis; median years in the SP 7.7 years; AC 7 years). There were fewer females in the AC group (63:183) with a male to female ratio of 1:2.9 compared with 1:2.3 among the SPs (51:116) but this was not significant (p = 0.27).

**Table 1:** Characteristics of the HTLV-1 cohort and the indications for CT imaging

	Symptomatic patients	Asymptomatic carriers
Ethnicity		
African/Afro-Caribbean	139	161
Caucasian	21	69
Asian	2	10
Japanese/Chinese	1	4
South American	2	1
Not known	2	1
M:F ratio	51:116	1:2.9 p = 0.27
Median HTLV proviral load at 1^st^ visit (HTLV DNA copies/100 PBMC)	15.9 %	1.25 % p < 0.0001
Lymphoma/Leukaemia	29.3	
HAM/TSP	14.0	
HAID	8.5	
Strongyloidiasis	20.6	
Time from HTLV diagnosis (years) to CT	2	2.5
Indication for CT imaging (bronchiectasis)		
Productive cough +/- recurrent chest infection	15 (9)	17 (1)
Weight loss	7	9
Suspected malignancy	2	8
Abnormal chest X-ray	5 (4)	
Unclear	1	

HTLV-1 VL, which was available for 167 SPs and 234 ACs, was significantly higher in SPs (median HTLV VL 15.9 % 95 % CI 13.7 – 20) compared with ACs (median 1.25 %; 95 % CI 0.82 – 1.69, p <0.0001). HTLV-1 VL among non-HAM/TSP SPs was higher still (median 21.6 %, non-HAM/TSP vs. AC; p = 0.0001). The median VLs in the different SP groups were: HAM/TSP 14 %, HAID 8.5 %, ATLL 29.3 % and Strongyloidiasis 20.6 % (Table 1).

Thirty four of 246 ACs and 30/167 SPs had a CT chest. Of the CT scans performed, 4/34 (11.7 %) in the AC group and 10/30 (33.3 %) in the SP group were high resolution CTs. (HRCTs). The reasons for CT imaging were productive cough +/- recurrent infection (50 % ACs vs. 50 % SPs), weight loss (26.5 % ACs vs. 23.3 % SPs), suspected malignancy (23.5 % ACs vs. 6.7 % SPs) or an abnormal chest X-ray (SPs 16.7 %). The indication for CT was unclear in 3.3 % SPs. The indications for HRCT was productive cough +/- recurrent infection. The time from HTLV-1 diagnosis to imaging was on average 2.5 years in ACs and 2 years in SPs (Table 1). Out of the total 64 patients that underwent CT imaging, 13 were Caucasian (13/90, 14 %) and 51 were Afro-Caribbean (51/300, 17 %).

Fourteen patients were newly diagnosed with bronchiectasis based on HRCT, (indications detailed in Table 1): 1/246 AC (0.4 %) and 13/167 SPs (2 with HAID, 1 with ATLL, 10 with HAM/TSP) (7.8 %, RR 19.2 95 % CI 2.5-14.5, p = 0.004). Bronchiectasis was more common in those with HAM/TSP compared to non-HAM/TSP patients (10/95 vs. 4/318, RR 8.4 95 % CI 2.7-26.1, p = 0.0002). None of our patients had a history of significant childhood infections as per the patient or their health records. We thereby did not identify the other causes of bronchiectasis including congenital conditions, tuberculosis, exposure to chemical irritant or childhood infections. There were also no documented or clinical features to suggest a diagnosis of bronchiectasis at or prior to the diagnosis of HTLV-1 infection as per the patient or their health records. We did not exclude alpha-1 antitrypsin deficiency or autoimmune conditions however our patients had no other clinical symptoms or signs to suggest these conditions. There was also no history of organ failure or myocardial disease. All of our patients with bronchiectasis were non-smokers. Through detailed history obtained from the patients and their health records, they were diagnosed with bronchiectasis 1-3 years following their initial HTLV-1 diagnosis and hence at least 1-3 years from any initial symptoms of HTLV-1 infection.

The characteristics of the patients with bronchiectasis are summarised in Table 2. The median HTLV-1 VL at the time of diagnosis of bronchiectasis was 25.2 % in the SPs and 3.1 % in the solitary AC. Although higher, the median VL in HAM/TSP patients with bronchiectasis was not significantly different from the remaining HAM/TSP patients (25.2 % vs. 14 %, p = 0.72). Bronchiectasis was more common amongst Caucasians (6.7 %; 6/20 SPs & 0/70 ACs) than African/Afro-Caribbean (2.0 %; 5/137 SPs & 1/163 ACs, Fisher’s exact test p = 0.01). The relative risk of bronchiectasis from not being African/Afro-Caribbean was 3.45 (1.2-9.7, p = 0.02). There was no significant difference in age at presentation to the HTLV clinic diagnosis of HTLV-1 between SPs with and without bronchiectasis (median 59.3 vs. 53.9 years, p = 0.43) nor between the patients with HAM/TSP with and without bronchiectasis (median 59.5 vs. 54.7 years, p = 0.48).

**Table 2:** Characteristics of the HTLV-1 patients with a formal diagnosis of bronchiectasis

	Symptomatic patients	Asymptomatic carriers
Number of patients	13	1
Median age at 1st presentation (years)	59.3	50.9
M:F	5/8	1 female
Ethnicity		
African/Afro-Caribbean	5	1
Caucasian	6	
Asian	1	
South American	1	
Median HTLV-1 proviral load at diagnosis of bronchiectasis	25.2 %	3.07 %
Bilateral: unilateral bronchiectasis	11/2	1/0
Cylindrical: cystic bronchiectasis	11/2	1/0

In the multivariate analysis disease state (p < 0.001) and ethnicity (p = 0.02) but not age, gender or HTLV-1 VL were independent predictors for bronchiectasis.

## Discussion

Our data suggest that bronchiectasis is more common among HTLV-1 infected subjects in the UK than among the general population, that the inflammatory process of HAM/TSP and other HAID is implicated in pathogenesis and that Caucasians infected with HTLV-1 may be more susceptible to bronchiectasis than African/Afro-Caribbeans.

Mass chest radiography during the 1950s indicated a prevalence of 100 per 100,000 adult UK population of bronchiectasis [30]. The 3.4 % documented prevalence of bronchiectasis in our HTLV-1 cohort is higher than that reported in general UK (0.1 %) or US populations (0.04-.0.27) [30, 31].

In the general population the prevalence of bronchiectasis increases with age. A retrospective cohort study in the US found the prevalence of bronchiectasis to be 4.2 per 100,000 population aged 18-34 years and 271.8 per 100,000 population aged >75 [30]. However age was not an independent variable in this cohort, nor is the cohort particularly old (median age 48.6 years).

Another consideration is the ethnic bias of the cohort which is predominantly of African/Afro-Caribbean ethnicity. However not being African/Afro-Caribbean carried a greater than 3 fold increased risk for diagnosed bronchiectasis. Cohort data from the two Caucasian populations with highest HTLV-1 prevalence, Iran and Romania have not been reported. Whilst the lower risk of bronchiectasis observed in African/Afro-Caribbean subjects merits further investigation this may account for the paucity reports of association between HTLV-1 and bronchiectasis from outside of Japan. Genetics, timing of primary infection and environmental factors may all contribute to these different rates. Difference in bronchiectasis rates amongst different ethnicities have been reported from New Zealand and Fiji between Polynesian and Non-Polynesian and Melanesian and Indian children respectively [32, 33]. Whilst the underlying cause of bronchiectasis has also been shown to be influenced by ethnicity [34].

We hypothesised that HTLV-1 VL would be associated with bronchiectasis and as previously reported for this cohort, and consistent with other studies, HTLV-1 VL was significantly higher in patients with symptomatic HTLV-1 infection than in the ACs [18]. However although HTLV-1 VL was high in all the patients with bronchiectasis, including the subject previously characterised as AC, an association between bronchiectasis and high HTLV-1 VL was not found in the multivariate analysis whereas pre-existent disease was strongly associated suggesting that prior inflammatory disease (HAM/TSP or HAID) rather than VL per se was the key association. This theory is further supported by the median VL in the SPs with bronchiectasis not being significantly higher than the VL reported for all SPs (25.2 % SPs with bronchiectasis, 15.90 % all SPs p = 0.62), even when the patients with ATLL (who generally have the highest VLs) were excluded (14 %; p = 0.067). This suggests that whilst high VL may be essential it is not sufficient for the development of bronchiectasis. This is further supported by the association of bronchiectasis with HAM/TSP rather than ATLL pointing to a key role of inflammation rather than immune suppression in the aetiology, although both may co-exist and the course of ATLL may be too short or too severe to result in bronchiectasis. However, since ATLL is thought to follow many decades of HTLV-1 infection, occurs later than HAM/TSP and against a background of pre-existing high VL this again points to inflammation secondary to high VL, rather than VL per se in the pathogenesis of HTLV-1-associated bronchiectasis.

Our HTLV-1 cohort is comprised of patients from the three main categories of infection, namely asymptomatic carriage, ATLL and HAM/TSP and, as reflects the demographics of HTLV-1 infection in the UK, was predominantly of African/Afro-Caribbean ethnicity. The high number of patients that were Caucasian in the AC group is secondary to universal blood donor screening. The M: F ratio between ACs and SPs was similar. A major cause for bronchiectasis is early childhood infections. This can be difficult to exclude however there was no significant childhood infections reported by the patient or in their health records. Further to this even though we did not test for autoimmune conditions or alpha 1 antitrypsin deficiency our patients had no clinical symptoms or signs of these conditions.

The pathogenesis of bronchiectasis associated with HTLV-1 has not been studied. The association here with a prior diagnosis of another inflammatory disease and the prominent lymphocytosis in BAL reported by others [19, 21] points to the same inflammatory state that causes HAM/TSP. Another possibility is that HTLV-1 causes a degree of local immune suppression (perhaps akin to infective dermatitis) which makes the patient prone to low grade pulmonary infections which in turn predisposes them to complications particularly bronchiectasis. The most striking example of HTLV-associated immune impairment is seen in the relationship between HTLV-1 infection and *Strongyloides stercoralis,* with a higher incidence HTLV-1 amongst *S. stercoralis* carriers [35], and a higher incidence of strongyloidiasis in HTLV-1 seropositive patients than in HTLV-1 negative individuals [36].

### Strengths and limitations of this study

The major limitation of this study is is that CT imaging had only been performed if the patient had clinical or radiological features that raised the suspicion of underlying pulmonary disease with no lower threshold for imaging in this cohort compared to the general population. Thus only 15 % of the cohort had CT imaging (13.8 % ACs and 18.0 % SPs) and the prevalence of bronchiectasis, particularly early/mild or asymptomatic, may have been underestimated. Second, the number of cases of bronchiectasis is relatively few. Despite this the associations reported are statistically robust, suggesting that these are not chance findings. Third, we have no data on the effect of HTLV-1 or associate inflammatory cells in the lung and can only speculate regarding pathogenesis. The relative strengths are studying a population that is socially and ethnically distinct and the median seven years follow-up of each patient group. A prospective study is now justified to confirm and extend these findings on the frequency of bronchiectasis in patients with HTLV particularly amongst those with pre-existing HTLV-associated disease and to explore pathogenesis.

## Conclusion

In the UK bronchiectasis is more common amongst HTLV-1 infected subjects (3.4 %) than reported for the general population (0.1 %). Non-African/Afro-Caribbean patients with pre-existing HTLV-1-associated disease are most at risk. Our data support the view that the spectrum of HTLV-1-associated disease is broader than previously thought and that disseminated mild inflammation may be common, albeit asymptomatic. Our results emphasise the importance of screening symptomatic HTLV-1 patients for pulmonary disease (i.e. radiological imaging) early following diagnosis of HTLV-1 and that CT imaging should be requested even in the presence of minimal symptoms. Furthermore HTLV serology should be considered in all patients with bronchiectasis in whom no other cause is apparent.
